# 

**Published:** 1899-03

**Authors:** 


					MARCH, 1899.
THE
AMERICAN JOURNAL
?w- O F -w-
DENTAL SCIENCE.
EDITED BY
F. J. S. GORGAS, ]tf: D.n D. P, S.
RICHARD
Vol. 38.
THIRD StlH-E S--
So. It.
BALTIMORE:
SNOWDEN 4. COWMAN MFG. CO., 9 W. Fayette St.
LONDON:
TRUBNER A, CO., 60 Paternoster Row.
Entered at the Post Office at Baltimore, Md., as Second-class Matter.
PRYHBLE IN HDVRNCE.
IPUBLISHED MONTHLY.
$2.50 PER flNNUMJ

				

